# Ti_3_C_2_T_x_-Based Materials and Coatings for De-Icing and Defogging of Wind Turbine Blades: Materials Basis, Structural Design, Engineering Integration, and Future Opportunities

**DOI:** 10.3390/nano16120784

**Published:** 2026-06-22

**Authors:** Weiwei Wu, Kening Peng, Kunqi Zhang, Zhifang Liu, Nana Yao

**Affiliations:** 1 Sinoma Wind Power Blade Co., Ltd., Beijing 100192, China; 2 Institute of Atomic Manufacturing, Beihang University, Beijing 100191, China; 3 School of Environment and Chemical Engineering, Foshan University, Foshan 528000, China

**Keywords:** MXene, wind turbine blade, de-icing, defogging, coating architectures

## Abstract

In cold, humid environments, even a small amount of ice accumulation on the blade surface can degrade aerodynamic performance, increase drag, induce premature stall and vibration, and raise the risks of shutdown, fatigue, and ice throw. Existing blade anti-icing and de-icing strategies (such as passive coatings, electrothermal heating, hot-air systems, and hybrid designs) struggle to simultaneously meet the requirements of lightweight construction, low-voltage rapid heating, conformability to curved surfaces, erosion resistance, long-term durability, and scalable manufacturing. MXenes, particularly Ti_3_C_2_T_x_, have attracted attention due to their high electrical conductivity, broadband optical absorption, solution processability, tunable interfacial chemistry, and good compatibility with polymer matrices. However, their oxidation issue and blade-scale deployment challenges (coating chemistry, scalable fabrication, real-world testing) remain obstacles. Based on this, this review discusses Ti_3_C_2_T_x_-based anti-icing, de-icing, and defogging strategies for wind turbine blades, with emphasis on material properties, functional mechanisms, coating architectures, fabrication routes, durability, and scalability, and highlights their potential for lightweight and energy-efficient all-weather blade protection. Finally, future research directions for Ti_3_C_2_T_x_-based blade anti-icing and de-icing are prospected. This review not only aims to identify key knowledge gaps in current research but also strives to provide a theoretical reference for the application of Ti_3_C_2_T_x_ in the complex service environment of real wind turbine blades, thereby moving beyond idealized laboratory conditions.

## 1. Introduction

### 1.1. Background and Challenges in Blade De-Icing

Wind energy plays a key role in the global transition to low-carbon power systems, but in cold and humid regions, its reliability still depends heavily on how blades respond to icing and persistent condensation. Among all turbine components, the blade is the most aerodynamically sensitive [1,2]. A relatively thin and rough ice layer near the leading edge can already distort the local pressure field, reduce lift, increase drag, trigger premature stall, and ultimately lower annual energy yield [3,4,5,6]. The blade is therefore not just a substrate for coating deposition. Its geometry, composite structure, and aerodynamic sensitivity directly determine whether an anti-icing layer can work in service. De-icing a utility-scale blade is much harder than melting ice on a flat laboratory coupon [7]. Real blades are large curved composite structures with nonuniform thickness, embedded reinforcing fibers, spatially varying thermal mass, and constant exposure to airflow, rain erosion, ultraviolet irradiation, and cyclic mechanical loading. Any practical anti-icing or de-icing strategy must therefore meet several requirements at once: low added mass, minimal aerodynamic penalty, compatibility with composite manufacturing and repair, modest power consumption, large-area conformability, robust adhesion, and tolerance to freeze–thaw and erosive service [8,9]. Many concepts that work well on flat metallic substrates become far less convincing once transferred to meter-scale blade surfaces, where boundary conditions, damage modes, and integration costs are much more demanding.

Condensation and fogging are often treated as secondary issues, but they can strongly affect the early stages of icing [10]. When the blade surface approaches the dew point, droplets can nucleate, spread, and coalesce into water films or dense microdroplet layers. These condensed phases increase surface wetness, alter effective droplet mobility, change local heat transfer, and often accelerate the transition from delayed icing to frost or glaze. In heating-based systems, they also consume latent heat and aggravate thermal nonuniformity. A practical blade surface should not only remove ice after formation but also suppress or rapidly clear condensate to interrupt the icing sequence at an early stage. Current strategies can be grouped broadly into passive, active, and hybrid approaches. Passive methods, such as low-surface-energy coatings, textured superhydrophobic surfaces, and slippery interfaces, aim to delay nucleation, reduce ice adhesion, or facilitate water shedding without continuous energy input [11,12]. Their appeal is obvious, but their durability under real blade service remains a persistent concern. Micro- and nanostructures are vulnerable to droplet impact and erosion, low-surface-energy species can migrate or degrade, and purely passive surfaces usually lose effectiveness once a continuous ice layer develops. Active approaches, including electrothermal heating, hot-air systems, mechanical vibration, pneumatic actuation, microwave heating, and electro-impulse methods, are better suited to removing established ice, but often at the cost of added complexity, higher energy demand, and uneven temperature fields. For wind turbines, the most realistic path increasingly appears to be a hybrid design, in which surface chemistry and structure slow the onset of icing while an efficient heater removes the remaining condensate, frost, or thin ice only when needed.

### 1.2. MXene (Ti_3_C_2_T_x_) as a Potential Solution and Review Scope

MXenes are well positioned within this design space. Since the emergence of two-dimensional transition-metal carbides and nitrides, especially Ti_3_C_2_T_x_, they have attracted sustained attention because they combine metallic conductivity, broad optical absorption, rich surface chemistry, and straightforward solution processing [13]. These attributes are highly relevant to blade protection. Compared with conventional polymer coatings, MXene-containing films can transport current efficiently and generate Joule heat over large areas at relatively low voltage [14]. Compared with metal-foil heaters, they can be made much thinner and more conformable. Compared with many nanocarbon-only networks, they offer more accessible surface functionality for bonding with polymers, primers, and composite substrates [15]. As a result, Ti_3_C_2_T_x_ are plausible candidates for distributed electrothermal layers, photothermal absorbers, multifunctional interlayers, and hybrid coatings that integrate heating, water management, sensing, and possibly electromagnetic functions [16]. Ti_3_C_2_T_x_ alone cannot serve as a direct solution for large-scale blade applications, and their oxidation behavior remains the biggest challenge to long-term use. A blade-facing MXene system must withstand oxygen, humidity, salt, ultraviolet radiation, dust, and repeated thermal cycling while retaining conductivity, adhesion, and coating integrity. Dense restacking may improve barrier properties but can sacrifice accessibility and flexibility, whereas overly open networks are more susceptible to environmental degradation and tend to form nonuniform current pathways [17,18]. Blade-scale implementation also requires more than good materials data: it depends on robust coating chemistries, scalable deposition routes, practical electrode architectures, and testing under realistic icing-wind conditions rather than only static bench-top demonstrations.

Here, this review emphasizes the practical requirements for applying MXene-based coatings to real wind turbine blades. We move beyond small-sample anti-icing demonstrations and discuss Ti_3_C_2_T_x_-based systems in the context of curved composite substrates, aerodynamic sensitivity, rain erosion, thermal nonuniformity, power management, and long-term durability. Table 1 summarizes the key design considerations discussed in this review.

## 2. Wind Turbine Blade Icing and Fogging: Service Scenarios and Engineering Constraints

### 2.1. Icing Modes, Atmospheric Conditions, and Consequences

Blade icing is not a single, uniform process. Rime ice usually develops at low temperature and relatively low liquid-water content, producing rough and porous deposits. Glaze ice forms when supercooled droplets spread before freezing, often leading to denser and more adherent layers. Mixed ice exhibits feature of both. Offshore turbines may also face saline aerosols, while mountainous installations commonly encounter cloud icing and strongly fluctuating thermal conditions. Accretion is likewise spatially heterogeneous: the leading edge is typically the most vulnerable region, but local flow separation, pressure gradients, roughness, and blade geometry can shift the deposition pattern along both span and chord. These distinctions are not merely descriptive; they directly influence materials design. Porous rime may be easier to detach mechanically, yet it sharply increases roughness and can promote further nucleation. Dense glaze imposes a larger thermal burden and often adheres more strongly. Frost and fog-derived ice may begin from water films or dense microdroplet coverage that spreads over a larger area than isolated impacting droplets. For this reason, a practical blade coating should not be tuned against one idealized icing mode only. It must address several stages of the process, from condensation control and rapid droplet removal to controllable heating once detachment becomes necessary. To quantitatively evaluate the ice accretion dynamics on a wind turbine blade, the local mass accumulation rate (R_acc_, kg·m^−2^·s^−1^) can be modeled by the mass balance framework [2]:

R_acc_ = β·LWC·v·f

where β represents the local droplet collection efficiency (a dimensionless vector governed by the droplet Median Volume Diameter (MVD) and the blade leading-edge radius), LWC is the atmospheric liquid water content (g·m^−3^), v is the relative airflow velocity(m·s^−1^), and f is the freezing fraction (0 ≤ f ≤ 1). Fundamentally, the thermodynamic phase transition at the coating–ice–air interface is dictated by the boundary-layer energy balance equation:

$$q_{ext}+q_{latent}=q_{conv}+q_{cond}+q_{sens}+q_{evap/sub}$$

where q_ext_ is the external active heat flux injected into the system (e.g., via MXene-enabled Joule heating or photothermal conversion), and q_latent_ = R_acc_·ΔH_f_ represents the latent heat released during solidification (ΔH_f_ = 333.5 kJ·kg^−1^). This heat input is counterbalanced by several cooling sinks: q_conv_ = h_c_ (T_s_ − T_a_) is the convective heat loss dominated by the local heat transfer coefficient (h_c_) under high Reynolds number airflows; q_cond_ is the conductive heat loss into the structural, anisotropic composite substrate; q_sens_ = β·LWC·v·C_w_(T_s_ − T_a_) defines the sensible heat required to warm the impinging supercooled droplets from ambient temperature (T_a_) to the surface temperature (T_s_); and q_evap/sub_ denotes the latent heat consumed by evaporation or sublimation [4]. When f = 1, all incoming droplets freeze instantaneously upon impact, resulting in porous rime ice typically observed at low temperatures and low LWC. Conversely, when f < 1, the unfrozen liquid runs back along the blade chord, forming a continuous water film that consolidates into high-density, strongly adherent glaze ice under higher LWC and near-freezing conditions (T_a_ ≈ 0 °C). This thermodynamic coupling underscores why rime, glaze, mixed ice, or frost cannot be evaluated purely through simplified static cold-plate tests; the convective heat transfer (q_conv_) and complex aerodynamic shear stress fields completely redefine the surface thermal requirements.

The aerodynamic penalties of icing are well established. As the blade surface becomes rougher and its profile is distorted, lift decreases, drag increases, and stall occurs earlier. The turbine power curve shifts, control actions become more conservative, and energy production falls. If ice accumulates unevenly, rotor imbalance increases cyclic loading on bearings and towers. Noise and vibration can intensify, sensor signals may become unreliable, and in severe cases, ice throw creates a direct safety hazard. Blade icing is therefore best understood as a coupled aerodynamic, structural, operational, and safety problem rather than a simple surface-freezing event.

### 2.2. Fogging and Condensate Effects Under Icing-Prone Conditions

Fogging is less dramatic than thick ice accretion, but it is highly relevant to the blade-surface energy balance. Condensed microdroplets change thermal and interfacial conditions, may spread into films under airflow, and often act as precursors to frost or glaze when temperatures drop further. Once such a film develops, textured passive surfaces can lose their non-wetting state, allowing liquid to penetrate surface features and sharply reducing apparent hydrophobicity. For active heating systems, the extra latent heat required to warm or evaporate this condensate becomes an additional energy penalty. In that sense, defogging is not a minor add-on; it is part of the anti-icing problem itself.

### 2.3. Requirements Specific to Wind Turbine Blades

A blade-oriented de-icing technology must satisfy requirements that are substantially stricter than those used in most laboratory demonstrations. Added weight must remain minimal, particularly at large radial positions where inertia and fatigue penalties increase. The heating strategy should work at moderate voltage and manageable current so that turbine-side power distribution remains practical. The coating must conform to large curved composite substrates and remain adherent under flexure, vibration, and thermal cycling. It must also tolerate rain erosion, sand impact, ultraviolet exposure, and, in some environments, salt spray. Just as importantly, it should not degrade blade roughness or compromise the protective leading-edge systems already used in service. Maintenance and repair must also be realistic under field conditions rather than dependent on factory-only processing.

A coating may exhibit excellent icing-delay time in static cold-plate tests yet fail under high-speed rain impact. A conductive film can heat rapidly on glass but adhere poorly to glass-fiber/epoxy composites. A hydrophobic topcoat may lower droplet pinning initially but degrade under ultraviolet irradiation or abrasion. Likewise, a photothermal material that performs under controlled illumination may contribute little during nighttime icing. For blade use, coating performance has to be evaluated together with substrate adhesion, heat loss, erosion, electrical layout, and repair conditions [7].

Consequently, traditional static cold-plate freezing delay or contact angle measurements are highly insufficient to validate field survivability. Comprehensive qualification requires simulation within certified icing wind tunnels (IWT), where the atmospheric parameters can be dynamically regulated to match standard icing classes (e.g., LWC spanning 0.1 to 2.0 g·m^−3^, MVD ranging from 15 to 50 μm, air temperatures down to −20 °C, and subsonic airflows matching real tip-speed ratios). Transitioning to these standardized multi-physical test frameworks is essential to bridging the translational gap for emergent 2D nanomaterial coatings.

### 2.4. Current Blade De-Icing Technologies and the Gap Ti_3_C_2_T_x_ May Fill

Among active strategies, electrothermal heating remains one of the most practical for wind turbine blades because it is controllable, mechanically simpler than pneumatic or impulse systems, and compatible with zonal activation. Existing heater concepts include metallic wires, conductive paints, carbon-based composites, and embedded resistive layers, but these often suffer from thickness, temperature nonuniformity, integration complexity, or high energy demand. Passive coatings can lower ice adhesion or delay freezing, yet their durability is often insufficient for service on large blades. Recent blade-focused studies increasingly point toward hybrid concepts in which a passive surface reduces the burden on a localized active heater.

Ti_3_C_2_T_x_ become relevant precisely in this hybrid design window. They can function as thin distributed heater networks, photothermal absorbers, reinforcing interlayers, or conductive nanosheets embedded within polymer-based coatings [19]. They can also be combined with fluorinated, silicone, polyurethane, epoxy, or elastomeric components to couple rapid Joule heating with tailored wettability and adhesion [20,21]. Translating the intrinsic properties of MXenes into blade-compatible architectures remains the central challenge.

## 3. Ti_3_C_2_T_x_-Based Coatings for Integrated De-Icing and Defogging

### 3.1. Family Characteristics and the Predominance of Ti_3_C_2_T_x_

**Figure 1:**
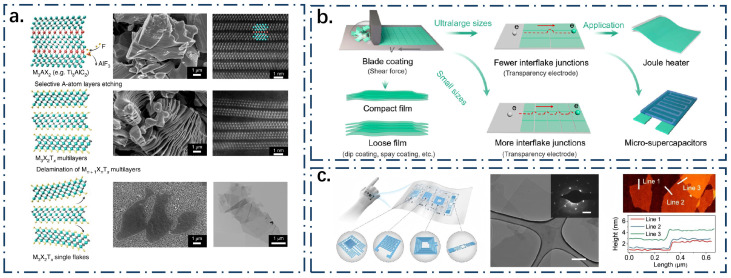
Multiscale route from Ti_3_C_2_T_x_ synthesis to structurally integrated coatings. (**a**) Top-down conversion of MAX phases into delaminated two-dimensional nanosheets [22]. (**b**) Scalable solution processing, exemplified by blade coating, for the fabrication of uniform large-area functional films [24]. (**c**) Flexible high-resolution Ti_3_C_2_T_x_ circuitry illustrating the mechanical compliance required for conformal integration on non-planar blade surfaces [28].

Ti_3_C_2_T_x_ are typically produced by selectively removing the A layer from MAX phases, yielding two-dimensional carbides, nitrides, or carbonitrides terminated with surface groups such as –O, –OH, and –F [22]. Among them, Ti_3_C_2_T_x_ has become the dominant model system because it can be synthesized reproducibly, dispersed in water or polar solvents, processed into films and coatings, and integrated into a wide range of composites. For anti-icing and defogging applications, this level of maturity is critical for blade-oriented applications. Ti_3_C_2_T_x_ combines high electrical conductivity with strong broadband optical absorption, while still offering chemically active surfaces for bonding, crosslinking, and dispersion control [23,24]. The synthesis-to-coating transition sketched in Figure 1a,b helps explain why Ti_3_C_2_T_x_ is currently the most realistic MXene platform for blade-oriented studies.

This emphasis on Ti_3_C_2_T_x_ does not mean the rest of the MXene family is irrelevant. Other compositions may offer different oxidation behavior, thermal properties, or spectral response [25,26,27]. However, when the target is near-term blade integration rather than exploratory materials discovery, Ti_3_C_2_T_x_ still has the clearest practical advantage: its processing protocols, dispersion chemistry, film fabrication methods, and composite formulations are far more developed than those of less-studied MXenes [26].

### 3.2. Electrical Conductivity and Distributed Joule Heating

The most immediate attraction of Ti_3_C_2_T_x_ for blade heaters is their conductivity. Well-assembled Ti_3_C_2_T_x_ films can reach sheet resistances suitable for large-area Joule heating at moderate voltage, even when deposited from solution or incorporated into polymer matrices. Relative to particle-filled polymers, MXene nanosheets can establish percolating in-plane transport pathways at lower loading because of their large aspect ratio and planar overlap [28]. Relative to wire-based heaters, they distribute current more continuously across an area rather than along discrete lines. That characteristic is advantageous for blade surfaces, where hotspots and cold stripes are both undesirable.

Conductivity by itself is not the whole story. A usable blade heater also requires a resistance window that matches available power electronics, stable output over repeated cycles, and tolerance to local defects, strain, and cracking. MXene networks can be tuned through flake size, loading, orientation, interflake distance, hybridization with nanocarbons, and polymer confinement [29,30]. In practice, the goal is not always the lowest possible resistance. Networks with excessively low resistance may require high currents at fixed voltage, complicating power management, whereas a carefully optimized resistance range can provide more practical heating behavior at the system level.

### 3.3. Broadband Optical Absorption and Photothermal Contribution

Ti_3_C_2_T_x_ also exhibit strong broadband optical absorption and can therefore contribute to photothermal heating [31]. For wind turbine blades, photothermal action is unlikely to replace electrothermal control because many icing events occur under weak illumination or at night. Its value is more subtle. Under daylight conditions, photothermal absorption can reduce the extra electrical input needed to keep a critical blade region above the freezing threshold or to accelerate post-icing recovery. Even a modest temperature rise can alter droplet freezing dynamics when the coating is thin and the thermal margins are narrow.

The importance of photothermal design is not limited to energy savings. Light-absorbing surface or subsurface layers can be integrated with low-ice-adhesion or wettability-engineered interfaces to create hybrid systems with more than one route to performance. In this regard, Ti_3_C_2_T_x_ are attractive because the same phase that provides Joule heating can also contribute to optical absorption, simplifying the formulation relative to multi-additive designs [32]. Recent anti-icing studies increasingly reflect this shift toward dual-mode or multimode architectures.

### 3.4. Surface Chemistry, Adhesion, and Composite Compatibility

Unlike comparatively inert graphitic fillers, Ti_3_C_2_T_x_ carry abundant surface terminations that strongly affect how they interact with polymers, primers, and substrates [33]. This chemistry is both an advantage and a liability. On one hand, it enables hydrogen bonding, ionic interactions, and covalent functionalization, which supports dispersion in polar media and strong coupling to surrounding matrices [34,35]. That is highly beneficial in blade coatings, where adhesion to epoxy-, polyurethane-, or composite-based surfaces often governs service life more than intrinsic nanofiller properties do. On the other hand, the same reactive surfaces contribute to oxidation and environmental instability under humid and oxygen-rich conditions [36,37,38].

From an engineering standpoint, this tunable interfacial chemistry is one of the strongest arguments for using Ti_3_C_2_T_x_. Blade coatings are seldom single bare films. They usually consist of a primer or tie layer, a matrix or binder, optional hydrophobic modifiers, protective top layers, and sometimes gradient interfaces. Ti_3_C_2_T_x_ can participate in several of these roles at once: as conductive nanosheets within a binder, as interfacial coupling agents, as photothermal fillers, or as lamellar phases that lengthen diffusion pathways for water and oxygen.

### 3.5. Mechanical Compliance and Solution Processability

Large turbine blades require coating routes that are inherently scalable, such as spray deposition, blade coating, doctor blading, slot-die coating, dip coating, roll-to-roll lamination, or related industrial processes. Ti_3_C_2_T_x_ are attractive in this respect because they can be formulated as inks and dispersions rather than requiring vacuum deposition or high-temperature processing incompatible with polymer-composite blades [36]. That same solution processability also favors repair workflows, in which a damaged region may need local recoating or patching rather than full replacement.

Mechanical compliance is equally important. A highly conductive layer is of limited use if it cracks during blade flexure or thermal cycling. Dense MXene films alone can be relatively fragile out of plane, but when combined with elastomers, polyurethane, epoxy, or hybrid carbon scaffolds, they can form conductive layers with better crack tolerance and structural compliance [28,39]. Figure 1c illustrates conformal electronics on non-planar surfaces require conductivity and mechanical adaptability to be designed together rather than separately.

### 3.6. The Central Limitation: Oxidation and Environmental Degradation

The central durability issue is oxidation. Progressive oxidation reduces conductivity, changes surface chemistry, introduces structural defects, and can ultimately convert Ti_3_C_2_T_x_ into much less conductive oxide-rich products [40]. For blade applications, this matters even more than in many laboratory demonstrations, because the environmental exposure is severe: humidity, oxygen, salt spray, ultraviolet radiation, dust, and repeated heating cycles can all accelerate degradation. In a distributed heater, even moderate nonuniform degradation can create dangerous thermal inhomogeneity, local overheating, or complete loss of function.

Mitigating this vulnerability requires a multidimensional approach centered on environmental stability. First, synthesizing high-quality, large-lateral-size flakes with minimal in-plane defects and edge-to-basal-plane ratios can significantly slow oxidation kinetics from the source [34]. Second, incorporating antioxidant additives (such as ascorbic acid) into MXene dispersions or applying partial covalent passivation to the terminal groups can effectively scavenge reactive oxygen species and reduce the density of reactive surface sites [40]. Third, macroscopic architectural design is essential. Embedding MXene within low-oxygen-permeability polymer matrices creates tortuous diffusion pathways that restrict moisture and oxygen ingress [39]. Furthermore, the multilayer stack design, in which the MXene layer is buried beneath a thermally conductive, erosion-resistant, and moisture-barrier topcoat, effectively decouples environmental protection from electrothermal performance without introducing excessive thermal resistance.

In sum, stability should be treated as a primary design axis rather than a secondary materials issue. Storage conditions, flake quality, chemical post-treatment, antioxidant additives, barrier layers, polymer encapsulation, and multilayer stack design all need to be considered from the outset. Furthermore, given the long service intervals expected for wind turbine blades, accelerated aging evaluations and lifetime predictions are indispensable for ensuring credible field deployment.

### 3.7. Lightning-Strike Compatibility of Ti_3_C_2_T_x_-Based Coatings

Among these integration challenges, lightning-strike compatibility deserves particular attention because wind turbine blades are among the most lightning-exposed structures in service. Modern blades incorporate dedicated lightning protection systems (LPS) comprising receptors, internal down-conductors [41], and grounding paths. The introduction of a conductive Ti_3_C_2_T_x_-based coating or heater layer near the blade surface can alter local surface-potential distribution and may modify current pathways during a lightning attachment. A continuous, highly conductive MXene film covering a large fraction of the blade could, in principle, compete with designated receptors as an attachment point or divert a portion of the lightning current along unintended paths, potentially causing coating delamination, localized vaporization, or damage to the underlying composite structure [42].

The high conductivity of Ti_3_C_2_T_x_ (up to 2.4 × 10^4^ S·cm^−1^) makes it a promising candidate for both Joule heating and lightning-strike protection. In a recent experimental study, a Ti_3_C_2_T_x_-divinylbenzene coating subjected to a 100 kA simulated strike reduced the damage area on a carbon fiber composite panel from 40.61 cm^2^ to 13.29 cm^2^, and retained more than 78% of its residual flexural properties [17]. Compared to conventional copper meshes, which add significant weight and are prone to corrosion, MXenes offer comparable specific conductivity together with surface functional groups that bond well with polymer matrices. Nevertheless, applying such conductive coatings to a turbine blade requires careful electrical coordination with the existing LPS. Practical design measures include segmenting the MXene layer into isolated heating zones, burying the heater beneath an insulating topcoat that still allows thermal coupling, and tuning the sheet resistance so that the coating does not compete with the primary down-conductors. High-voltage impulse testing under standards will be needed to verify that these hybrid architectures can survive a lightning strike without compromising either the heater function or the blade’s structural integrity.

### 3.8. Comparative Benchmarking Against Alternative Conductive Heaters

To establish a clear engineering baseline and accommodate standard single-column journal formatting, Ti_3_C_2_T_x_ is critically benchmarked against alternative technologies via two structurally decoupled matrices: Table 2 evaluates emerging low-profile nanomaterial coatings, while Table 3 deconstructs macro-scale conventional and polymer-based heating elements.

Nanocarbon Frameworks (Graphene and CNTs): Compared with Ti_3_C_2_T_x_, graphene and carbon nanotubes (CNTs) possess vastly superior thermodynamic and chemical stability owing to their inert carbon basal planes (Table 2). However, their chemically hydrophobic nature presents processing challenges, leading to higher electrical percolation thresholds and a tendency to embrittle the polymer binder at high loadings.

Silver Nanowires (AgNWs): AgNW networks offer exceptional intrinsic metallic conductivity but suffer from acute cross-junction contact resistance (Table 2). They are highly vulnerable to thermal electromigration and ambient sulfidation under severe outdoor service, which induces severe resistance drift under cyclic mechanical strain.

Macro-scale Conventional Heaters (Carbon Fiber and Metal Foils): Carbon fiber heating blankets and embedded metallic meshes represent the current industrial standard (Table 3). While highly robust, they introduce severe weight penalties and local aerodynamic distortions. More importantly, metallic wires deliver discrete, localized “hot-line” heating, creating high spatial thermal gradients that accelerate localized microcracking and matrix debonding due to coefficient of thermal expansion (CTE) mismatches [42].

The Distinct Niche of Ti_3_C_2_T_x_: The unique value proposition of Ti_3_C_2_T_x_ lies in its fluid processing behavior (water-based inks) [15,34] and hydrophilic surface terminations -O, -OH, and -F, which enable strong interfacial cross-linking with polyurethane composite primers. It bridges the gap between rigid metals and poorly conductive polymers [14], offering a highly conformable, multi-mode (electrothermal-photothermal) hybrid coating architecture.

## 4. Functional Mechanisms: Electrothermal Heating, Photothermal Conversion, and Defogging Pathways

### 4.1. Electrothermal De-Icing

Electrothermal de-icing relies on Joule heating generated as current passes through a resistive network. For distributed coatings, the relevant material parameters include sheet resistance, current-carrying capability, thermal diffusivity, interfacial thermal resistance, and cycling stability. MXene-based heaters are attractive because thin films or coatings can reach useful temperature rises within short times at relatively low thickness. In many reported systems, the surface can be driven tens of degrees above ambient within seconds to minutes, depending on substrate, resistance, input voltage, and convective loss. Quantitatively, the generated steady-state areal power density (Pareal, W·cm^−2^) inside the Ti_3_C_2_T_x_ layer is governed by Joule’s law: Pareal = V^2/^(R_sheet_·A), where V is the applied voltage, R_sheet_ is the sheet resistance, and A is the heating area [43]. To evaluate the energy efficiency, the dimensionless electrothermal efficiency (η_et_) is introduced as η_et_ = q_interface_/P_areal_ = q_interface_/(q_up_ + q_down_), where qinterface represents the net heat flux reaching the target ice/coating boundary, while q_up_ and q_down_ denote the divergent heat losses escaped to the convective airflow environment and leaked into the back-end blade core, respectively.

For wind turbine blades, the practical objective is often not complete melting of thick ice over the entire surface. A more energy-efficient route is interfacial de-icing, in which the temperature at the blade–ice interface is raised just enough to weaken adhesion so that aerodynamic or gravitational forces remove the ice. That strategy dramatically lowers energy consumption relative to bulk melting. MXene coatings are well suited to this concept because they can be positioned as thin, near-surface heater layers close to the interface [44,45]. If combined with a low-ice-adhesion topcoat, the required temperature rise may be reduced further.

Uniformity is critical. Spatial variation in resistance leads to hotspots, localized thermal expansion, coating damage, and adjacent cold areas where ice persists. On centimeter-scale laboratory samples, such variations can be tolerable [46]. On blade-scale surfaces with long current paths, however, thickness fluctuations, flake agglomeration, cracking, and contact-resistance problems become far more consequential. Processing routes that control thickness and current distribution are therefore just as important as intrinsic MXene conductivity.

### 4.2. Photothermal Anti-Icing and De-Icing

Photothermal anti-icing works by converting absorbed light into heat, thereby delaying freezing or melting thin frost and ice. Because Ti_3_C_2_T_x_ absorb broadly across the solar spectrum, they have been incorporated into a growing number of photothermal anti-icing coatings [47]. On wind turbine blades, however, this mechanism is best understood as complementary rather than primary. It can prewarm the surface during daylight, shorten the residence time of condensed droplets, and assist recovery after intermittent icing, but it is unlikely to serve as the sole protection strategy in harsh climates.

The limitation is straightforward: solar energy input is variable and often unavailable when icing is most severe. A purely photothermal blade-coating strategy is therefore difficult to justify in practice. Electrothermal–photothermal synergy is more credible. During daylight, absorbed solar energy can reduce the electrical power needed to hold a blade section above a critical threshold; under low-irradiance or nighttime conditions, the electrothermal pathway remains available. When the same MXene phase contributes to both functions, the overall design can remain comparatively simple [48,49].

### 4.3. Passive–Active Synergy: Delaying Nucleation While Minimizing Power Consumption

A recurring lesson from recent anti-icing research is that synergy matters more than substitution. Passive features such as hydrophobicity, low ice adhesion, or tailored surface texture can delay freezing and reduce droplet residence time, while active heating can then be applied only intermittently or locally. This lowers the overall energy burden and widens the operating window. MXene-containing coatings fit this logic well because the conductive phase can be embedded within matrices that also control surface energy, elasticity, roughness, and barrier performance.

One practical configuration is a conductive MXene-rich subsurface layer combined with a top layer engineered for water repellency, low ice adhesion, or rapid condensate shedding [50]. Another is a hybrid composite in which fluorinated segments, silicone modifiers, or hydrophobic particles are introduced directly into the MXene-containing matrix. The difficulty, of course, is balancing conductivity with interfacial function: excessive hydrophobic or insulating additives can disrupt conductive percolation and cause nonuniform heating [51,52]. That tradeoff is one reason multilayer or gradient architectures are often more attractive than single-phase mixtures.

### 4.4. Defogging Mechanisms

The defogging mechanisms of MXene composite coatings rely on a synergistic combination of thermal regulation and surface engineering strategies. The primary mechanism is mild electrothermal heating, where the underlying MXene conductive network raises the surface temperature above the dew point. This approach is highly advantageous as it utilizes the same infrastructure intended for de-icing but operates at a significantly lower energy threshold. Additionally, the inherent photothermal properties of MXenes can absorb solar radiation during daylight, further reducing the electrical power consumption required to maintain clear surfaces under moderate humidity.

Beyond thermal regulation, surface wettability provides a complementary defogging pathway. By engineering the outermost protective layer to exhibit low contact angle hysteresis, condensed microdroplets can easily coalesce and detach under aerodynamic shear before forming continuous films [11,12]. In practical wind turbine applications, these mechanisms can be integrated into a multilayer architecture where the buried MXene layer delivers dual thermal functionalities, while the topcoat facilitates rapid condensate drainage. Furthermore, implementing zone-specific architectures allows for customized defogging strategies across different blade regions, offering an energy-efficient solution for early-stage frost suppression.

### 4.5. Heat Transport, Interfacial Resistance, and the Blade Substrate

The performance of any blade heater depends not only on how much heat is generated inside the coating but also on where that heat goes. Energy must be delivered efficiently to the ice-coating interface without excessive loss to airflow or the bulk substrate. To model this cross-thickness propagation, the multiscale coating stack can be simplified into a one-dimensional series thermal resistance network (R_th,total_) [5,43]:

$$R_{th,total}=\frac{d_{topcoat}}{k_{topcoat}}+R_{c}+\frac{d_{ice}}{k_{ice}}$$

where d and k denote the thickness and thermal conductivity of the respective topcoat/ice layers, and R_c_ represents the localized joint contact thermal resistance. Composite blades are anisotropic and comparatively poor conductors through the thickness, which can be advantageous because heat is retained near the surface. Primers, adhesives, and protective topcoats add thermal resistance. If the heater layer is buried too deeply, heat transfer to the surface becomes inefficient, increasing energy consumption; if it sits too close to the environment, it becomes vulnerable to erosion and moisture ingress. Crucially, rather than consuming massive energy for bulk ice melting, the system should target interfacial melting to induce a micro-scale liquid lubricating film, triggering an exponential decay in the absolute ice adhesion strength (τ_ice_):

$$\tau_{ice}=\tau_{0}\cdot \exp(-\alpha \cdot \Delta T)$$

where τ_0_ is the pristine structural adhesion capacity, α is a scaling coefficient, and ΔT represents the temperature elevation above 0 °C at the outermost interface (T_interface_ − 0 °C). This interfacial liquefaction breaks the mechanical anchoring, allowing aerodynamic lift and rotational centrifugal shear forces to cleanly shed the ice mass. In other words, anti-icing performance is determined not only by material choice but also by thermal architecture across the entire coating stack.

Beyond the basic heating mechanisms, overall blade performance is controlled by a set of interrelated design variables, from conductive filler selection to stack architecture and activation mode. These considerations are summarized in Table 4.

## 5. Materials Design Strategies for MXene-Based Blade Coatings

### 5.1. Pure MXene Films Versus Composite Architectures

**Figure 2:**
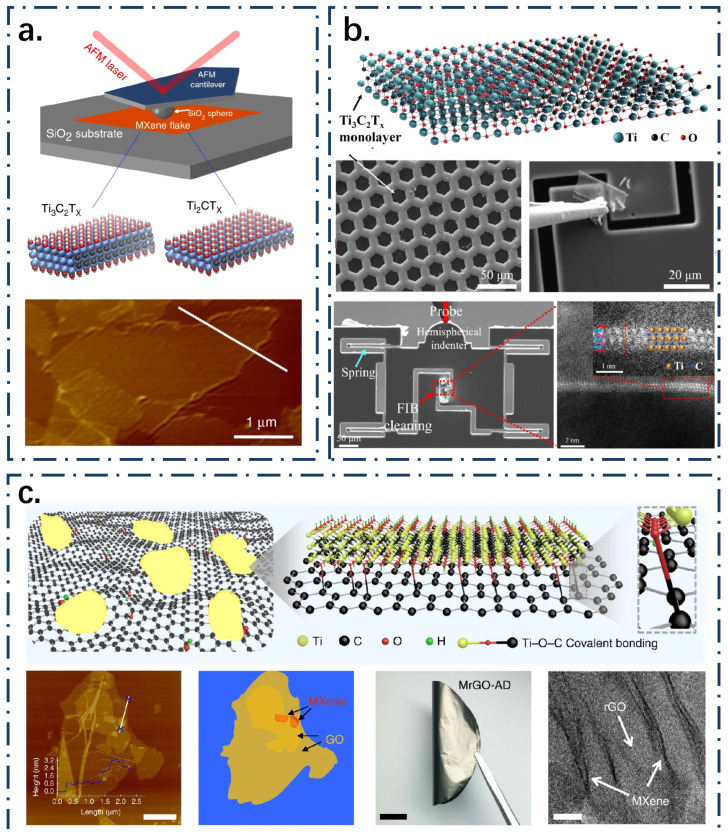
Interfacial engineering, mechanical robustness, and hybrid composite architectures of Ti_3_C_2_T_x_. (**a**) AFM methodology for evaluating the nanoscale adhesion properties of individual MXene flakes [56]. (**b**) Micromechanical testing setups and corresponding structural analysis demonstrating the intrinsic robustness and flexibility of MXene films [57]. (**c**) Hybrid structural designs, such as MXene networks integrated with reduced graphene oxide, engineered to enhance mechanical properties and ensure conductive network redundancy.

A bare MXene film can provide excellent conductivity and rapid heating, but for blade service, it is seldom sufficient by itself. Exposed films are vulnerable to oxidation, moisture, abrasion, and mechanical damage, and their environmental tolerance is rarely adequate for long-term outdoor use [53,54]. Practical blade-oriented designs, therefore, almost always involve composites, in which Ti_3_C_2_T_x_ are combined with polymers, elastomers, inorganic particles, or secondary conductive phases [55]. The matrix is not merely a passive carrier. It may act simultaneously as a binder, moisture barrier, stress-distribution medium, hydrophobic modifier, and erosion-resistant component. The central design challenge is to retain efficient conductive pathways while adding enough protection and processability for real service. In this context, the nanoscale interfacial adhesion between MXene flakes and the underlying resin can be quantified via atomic force microscopy (as shown in Figure 2a), while the overall structural integrity of the integrated system is validated through the micromechanical testing protocols illustrated in Figure 2b [56,57].

Polyurethane, epoxy, silicone, fluorinated polymers, and elastomeric binders are especially relevant because they are already familiar to the blade and coating industries. Polyurethane offers flexibility and weather resistance; epoxy provides strong adhesion and compatibility with composite substrates; silicone-based chemistries can lower surface energy and improve fouling resistance. Fluorinated modifiers are effective for water repellency but may bring cost or sustainability concerns. Which matrix is most appropriate depends on whether the MXene-containing layer is intended primarily as a heater, as a moisture-management layer, or as one component within a multifunctional stack.

### 5.2. Hybridization with Carbon Nanotubes, Graphene, and Carbon Black

Hybrid conductive networks can improve both performance and robustness. Carbon nanotubes, graphene derivatives, carbon black, and conducting polymers are often introduced to bridge MXene flakes, reduce restacking, and improve crack tolerance [58]. Carbon nanotubes are particularly effective as one-dimensional connectors between two-dimensional sheets, helping preserve conductivity under deformation. This synergistic effect is exemplified by the interleaved MXene/rGO hybrid architecture depicted in Figure 2c, which establishes a robust, redundant conductive network capable of maintaining de-icing performance under localized mechanical stress. Graphene and reduced graphene oxide can reinforce the structure and sometimes improve barrier properties, provided that dispersion and interfacial compatibility are well controlled [59]. Carbon black is attractive from a cost perspective, though excessive loading can raise viscosity and compromise coating uniformity [60]. For blade-scale applications, the value of hybrid networks is not limited to conductivity enhancement. They also provide redundancy. If part of the MXene network oxidizes or develops microcracks during service, a secondary conductive phase may preserve enough connectivity to avoid catastrophic heater failure [61,62]. In cyclic outdoor environments, that kind of graceful degradation is often more important than achieving the highest initial conductivity in pristine samples.

### 5.3. Hydrophobic and Low-Ice-Adhesion Modifications

Because icing begins with water arriving and remaining at the surface, many anti-icing designs combine conductive heaters with hydrophobic or low-adhesion surface chemistry. In MXene-based systems, this is usually achieved either by blending the conductive phase with hydrophobic additives and binders, or by applying a separate top layer. Fluorinated silanes, fluoropolymers, silicone segments, and roughness-generating nanoparticles have all been explored in the broader anti-icing literature [63,64]. The main difficulty is that the most water-repellent surface is not necessarily the most durable or the most thermally efficient.

For deployment, low ice adhesion is often more valuable than a very high static water contact angle alone [65]. Under realistic icing conditions, superhydrophobic textures may transition into fully wetted states because of pressure, repeated condensation, or wear. A mechanically durable coating with moderate hydrophobicity but low ice adhesion, combined with rapid interfacial heating, may therefore outperform a fragile surface that is initially superhydrophobic but quickly loses function. MXene-containing systems should be judged on the behavior of the full stack rather than on contact-angle data in isolation.

### 5.4. Encapsulation and Barrier Design for Oxidation Control

Encapsulation remains one of the most practical ways to slow MXene oxidation; protection may come from dense polymer matrices, lamellar barrier architectures, antioxidant additives, or multilayer stacks that reduce water and oxygen transport [66]. The difficulty is to shield the MXene without sacrificing the electrical and thermal transport required for heating. In some designs, the conductive network is buried beneath a thin protective topcoat; in others, the binder itself is engineered to provide both adhesion and barrier function [67]. Crosslinked polyurethane, epoxy, and hybrid organic–inorganic matrices are especially attractive because they can reduce permeation while remaining processable and mechanically robust [68,69].

Barrier design must also anticipate defects. Even a nominally dense coating can fail through pinholes, microcracks, exposed edges, or local damage near electrodes. Because blade surfaces operate in erosive environments, such defects are unavoidable over long service periods. That reality makes defect-tolerant strategies, such as crack-deflecting architectures, segmented heater layouts, or self-healing chemistries, more convincing than designs that assume a perfectly intact coating over years of operation.

### 5.5. Gradient and Multilayer Architectures

A useful design philosophy for blade coatings is to separate functions across the thickness of the stack. Asking one exposed layer to provide conductivity, adhesion, erosion resistance, ultraviolet stability, hydrophobicity, and low ice adhesion at the same time is rarely realistic. A multilayer architecture is more rational: a primer can be optimized for bonding to the composite blade, a MXene-rich layer can provide distributed heating, an intermediate layer can reduce moisture ingress, and an outer layer can be tailored for water management and erosion resistance [70]. Gradient structures can further reduce abrupt modulus or thermal mismatches between adjacent layers [71].

Ti_3_C_2_T_x_ are particularly versatile in such multilayer systems because they can function both as conductive phases and as lamellar barrier-building components [72]. Importantly, the MXene-rich layer does not need to be the outermost layer. Burying it beneath a protective topcoat may markedly improve lifetime, although at the cost of more complicated fabrication and stricter requirements on interlayer adhesion and thermal coupling. For industrial blade applications, the simplest architecture that still preserves function will usually be preferable to the most elaborate one.

### 5.6. Structural Design for Thermal Uniformity

Thermal uniformity is one of the most important yet underreported metrics in anti-icing coatings. It depends on electrical homogeneity, coating thickness, flake alignment, electrode layout, substrate coupling, and environmental convection. MXene flake size distribution matters as well. Larger flakes can reduce junction resistance, but may sediment more readily or create anisotropic defects; smaller flakes may disperse more uniformly but require more interflake contacts and thus higher overall resistance [73,74]. Large-area coating quality is therefore inseparable from rheology and process control.

Electrode design deserves equal attention. Even a well-formulated MXene coating will perform poorly if current injection is nonuniform [75]. Practical blade heaters may require busbars, segmented zones, or patterned electrodes tailored to local geometry and heat-loss conditions. Materials optimization and electrical design must therefore proceed together.

## 6. Fabrication and Integration Routes Compatible with Wind Turbine Blades

### 6.1. Solution Processing and Industrial Relevance

One of the strongest practical arguments for Ti_3_C_2_T_x_ is that they can be processed from liquid formulations [76]. Spray coating, dip coating, doctor blading, bar coating, slot-die coating, and printing-based methods are all, at least in principle, compatible with blade-oriented manufacturing. Yet industrial relevance depends on more than the ability to deposit a film. Ink stability, rheology, drying behavior, sedimentation resistance, and environmental sensitivity during processing are all critical. MXene dispersions may change with storage because of oxidation or aggregation, so formulation shelf life becomes a genuine manufacturing constraint [77].

Sprayable formulations are especially attractive for large curved surfaces and leading-edge regions, but spray deposition can also introduce thickness variations and overspray losses that directly affect heater uniformity and cost. Slot-die or bar coating can provide better thickness control, though these methods are harder to implement on strongly curved or already assembled blades. Lamination of prefabricated flexible heater films is another possible route, but it introduces additional interfaces whose adhesion and repairability must be carefully managed.

### 6.2. Direct Coating on Composite Substrates Versus Prefabricated Heater Laminates

Two broad integration strategies can be envisioned. In one, the MXene-containing system is deposited directly onto the blade or onto a primer or repair layer already present on the blade. This minimizes the number of interfaces and favors local repair, but the coating quality may depend strongly on substrate condition and field application environment [78]. In the other, the heater is fabricated first as a freestanding or supported flexible laminate and then bonded to the blade. That route offers tighter manufacturing control of the heater itself, but introduces a bonding interface that must remain durable under strain, moisture, and thermal cycling [79].

For large commercial turbines, direct in situ coating may be more attractive in retrofit or maintenance scenarios, whereas prefabricated laminates may suit factory-controlled manufacturing of new blades or replaceable modular heater strips at critical locations. The preferred option depends not only on materials performance but also on service philosophy and maintenance logistics.

### 6.3. Compatibility with Leading-Edge Protection Systems

Most modern turbine blades already include leading-edge protection systems to resist rain erosion. Any MXene-based anti-icing design must therefore coexist with those systems rather than be considered in isolation. This immediately raises a stack-design challenge. If the outermost layer must withstand high-speed droplet impact, the MXene-rich heater will often need to sit beneath a mechanically robust topcoat. That topcoat, in turn, must be thin and thermally efficient enough to allow useful heat transfer. An alternative is to introduce Ti_3_C_2_T_x_ directly into an erosion-resistant outer layer, but then, mechanical toughness cannot be sacrificed.

This coupling between anti-icing and erosion protection remains one of the clearest research gaps. Many MXene anti-icing studies report freezing delay or de-icing time but do not address rain erosion or leading-edge durability [80]. By contrast, blade-protection studies rarely consider MXene-enabled heating. Progress will likely depend on bringing these two research communities into closer contact.

### 6.4. Electrodes, Wiring, and Power Electronics

The performance of a blade heater depends as much on electrical architecture as on coating composition. Busbar design, contact resistance, insulation, segmented control, redundancy, and fault tolerance all shape the final temperature field. On long current paths, voltage drop can easily create thermal nonuniformity. Zonal layouts help address this issue by activating only the regions most vulnerable to icing, such as leading edges, root-transition zones, or sensor locations. MXene coatings are compatible with this approach because they can be patterned or selectively deposited [81].

Power-supply strategy is equally important. Turbine-side electronics must deliver stable voltage and current under changing environmental and operating conditions without imposing unacceptable parasitic loads. Smart control, guided by sensors or weather prediction, can markedly improve efficiency by activating heating only when condensate or icing risk is high. Here again, Ti_3_C_2_T_x_ may offer added value, since the conductive layer could potentially be integrated with self-sensing or companion sensing elements [82].

### 6.5. Repairability and Field Maintenance

Field-deployable MXene coatings need to be repairable on site. Minor damage should be restorable by localized surface prep, priming, and recoating, avoiding large section replacement. Two strategies are direct coating repair and modular laminate replacement. For direct coatings, a field repair sequence includes damage mapping, cleaning, mechanical removal, surface activation, repriming, recoating with MXene formulation, ambient curing, topcoat sealing, and electrical plus thermal verification, all executable at blade height on curved composites within weather windows. For laminate heaters, damaged sections are cut out, the surface prepared, a new MXene laminate bonded, and connections restored. This modular approach may be faster but needs spare laminates and reliable interconnects.

Future work should establish standardized repair protocols and quantitatively evaluate the recovery of electrical conductivity and adhesion strength in the context of MXene surface chemistry [52]. The durability of repaired zones under environmental aging, dynamic aerodynamic loads, and repeated repair cycles must be assessed, while shelf life and cure behavior in field conditions are characterized. Compatibility with existing leading-edge protection should also be verified, and overall performance validated through icing wind tunnel or rotating rig tests.

### 6.6. Scalability, Cost, and Supply Considerations

Even if MXene coatings prove technically effective, deployment on multi-megawatt turbines will require realistic control over cost, throughput, and supply. Etching chemistry, washing steps, delamination yield, storage stability, and waste treatment all contribute to the economics of MXene production [83,84,85]. For wind energy, reproducibility is often more valuable than record performance on a small coupon. A slightly less conductive but more durable and manufacturable coating may be the better engineering choice. Future studies should therefore report formulation stability, deposition rate, curing behavior, defect tolerance, and repair cost alongside conventional materials metrics.

To move toward a realistic deployment assessment, MXene-based blade coatings should be benchmarked against incumbent and emerging blade-heating technologies using several key techno-economic indicators. These include: (i) material cost per unit area, for which MXene-based systems depend strongly on synthesis route (HF, HF-free, molten-salt, or electrochemical etching) [15,34,83], precursor cost, delamination yield, and the number of washing and purification steps; (ii) coating areal loading or weight penalty (g/m^2^), which directly affects blade mass balance, fatigue loading, and the cost of any required counterweight adjustments; (iii) estimated areal power density (W/m^2^) required to achieve a given surface temperature rise under representative airflow, which determines the electrical infrastructure cost and the fractional turbine power consumption diverted to anti-icing [4,8]; (iv) balance-of-system costs, including busbars, wiring, connectors, power converters, and segmented control electronics; (v) formulation shelf life and pot life under field-relevant storage and application conditions; (vi) coating deposition throughput (m^2^/h) and compatibility with existing blade manufacturing or refurbishment workflows; (vii) anticipated repair cost and frequency over the blade service life; and (viii) supply-chain maturity, including availability of consistent-quality MXene feedstocks at multi-kilogram to ton scales [86]. Many of these indicators remain poorly quantified for MXene systems, and their determination under industrially representative conditions should be a priority for future blade-oriented studies. Based on the qualitative testing criteria for wind turbine anti–icing blades described above, a comparison of the strengths and weaknesses of different materials with respect to various performance metrics was conducted, and the findings are summarized in Table 5.

## 7. Performance Evaluation: What Should Be Measured, and How

### 7.1. Electrical and Thermal Metrics

Many reports still emphasize only the peak surface temperature reached at a fixed voltage, but blade-relevant evaluation requires a much broader set of metrics. These include sheet resistance, in-plane and through-thickness heat transport, heating rate, steady-state temperature under airflow, spatial temperature uniformity, response time, power density, and stability over repeated operation. Infrared thermography is useful, but it can be misleading if emissivity changes during icing or if only small coupons are examined. Large-area mapping and deliberately introduced defects are needed to reveal whether the heater remains uniform under realistic processing variability.

For electrothermal coatings, resistance drift is particularly important. A modest average change in resistance may reduce heating efficiency, but nonuniform drift is often more problematic because it can create hotspots and local failure. Accelerated aging should therefore track not only average electrical properties but also their spatial distribution and failure morphology.

### 7.2. Anti-Icing and De-Icing Metrics

Common anti-icing metrics include icing-delay time, droplet-freezing time, critical nucleation temperature, and ice-adhesion strength. De-icing studies often report de-icing time, melted-area fraction, temperature rise, or energy consumed per unit area. For wind turbine blades, however, interfacial de-icing energy and the threshold for ice shedding may be more meaningful than the time required for complete bulk melting. Test protocols should also distinguish among frost, rime, and glaze, since the relevant removal mechanisms differ substantially. Repeated icing/de-icing cycles are essential because coatings may degrade after only limited service exposure.

Airflow has a major influence on performance. A heater that appears effective in still air may behave quite differently in an icing wind tunnel or on a rotating blade because convective heat loss becomes much stronger. Thermal tests should therefore be coupled with controlled wind speed, droplet flux, and substrate geometry whenever blade deployment is the stated goal.

### 7.3. Defogging Metrics

Defogging lacks the rigorous evaluation given to icing. Useful metrics include how long it takes to clear condensate under a set humidity, how much droplet coverage remains, how droplets drain under airflow, and the smallest temperature rise that stops persistent fog. For opaque blade coatings, optical transparency matters little; what counts is how long wet films linger and turn to frost. Microscopy and airflow-assisted tests are more informative than transmittance measurements. To put defogging assessment on a firmer footing, blade-oriented coatings should regularly report a few key quantities. The condensate clearing time, measured by optical microscopy or high-speed imaging rather than transmittance, is essential because blade coatings are not see-through. The minimum temperature rise above ambient needed to suppress lasting condensation directly sets the power demand for continuous operation. Residual droplet coverage after a fixed heating period should be given as a function of humidity and airflow, since incomplete clearing can still seed frost. Drainage behavior under realistic airflow, including droplet motion, merging and detachment, matters because aerodynamic shear helps remove condensate on rotating blades. Finally, defogging stability must be checked after repeated humidity, icing and thermal cycles, as surface changes over time can degrade performance. These metrics allow fairer comparison across formulations and link defogging more directly to real blade conditions.

### 7.4. Mechanical Durability and Erosion

Mechanical durability is one of the decisive yet least consistently reported criteria for blade-oriented coatings. Leading-edge regions experience rain erosion, sand abrasion, and particulate impact, all of which can destroy surface texture, expose buried conductive layers, or fracture current pathways. Mild tape tests and simple abrasion tests are not enough. Blade-relevant assessment should include accelerated rain erosion, sand or slurry abrasion, cyclic bending, thermal shock, and freeze–thaw exposure, with electrical and thermal performance measured before and after testing. Equally important is whether damage is gradual or catastrophic.

### 7.5. Environmental Aging and Oxidation Monitoring

Because MXene oxidation is central to long-term performance, environmental aging must be built into the evaluation protocol rather than treated as an afterthought. Relevant exposures include humidity, liquid-water immersion, salt spray, ultraviolet irradiation, and thermal cycling [87,88,89,90]. Monitoring can involve sheet resistance, XPS or Raman analysis, optical absorbance, mass change, and microscopy of crack development. The point is not only to document decline but also to identify the dominant degradation pathway, whether it is edge oxidation, barrier failure, matrix embrittlement, or electrode corrosion.

### 7.6. Suggested Evaluation Hierarchy for Blade-Oriented MXene Systems

A staged evaluation pathway is helpful. Early screening can begin with flat coupons to assess conductivity, heating rate, basic icing delay, and adhesion. This should be followed by aged-coupon testing after humidity, UV, salt spray, and thermal cycling. The next steps are curved-substrate experiments under airflow with spatial temperature mapping, then integration with erosion-resistant stacks, and finally repeated icing-wind-tunnel tests on blade sections. Only after those stages should pilot-scale field validation be considered. Such a hierarchy helps prevent overclaiming based on early flat-plate results while still allowing efficient materials screening.

## 8. Lessons from the Current Literature

### 8.1. What the Broader MXene Anti-Icing Field Has Already Demonstrated

The broader anti-icing literature, even outside wind energy, has established several conclusions with reasonable confidence. MXene-containing coatings can provide rapid Joule heating at low thickness and moderate voltage [91]. Ti_3_C_2_T_x_ are also effective photothermal agents and can contribute meaningfully to hybrid active–passive designs. Polymer integration is usually necessary to achieve acceptable robustness and environmental protection [92]. In addition, water-management layers and low-ice-adhesion surfaces can lower the heating requirement, making hybrid systems more efficient than heater-only designs [93]. What remains less convincing in much of the literature is durability under harsh service, especially with respect to oxidation, abrasion, and long-term cycling [94].

For blade development, the main opportunity is therefore not to demonstrate once again that Ti_3_C_2_T_x_ can heat under an applied voltage. That point is already clear. The more meaningful task is to show that MXene-based stacks can retain that function on erosive, wet, ultraviolet-exposed, cyclically loaded composite surfaces while operating within realistic power budgets and maintenance constraints.

To establish a rigorous, data-driven foundation anchored in the verified literature, Table 6 cross-quantifies the authentic reported performance benchmarks and environmental aging parameters for representative Ti_3_C_2_T_x_ configurations, utilizing a transposed layout optimized for single-column page scannability.

Analysis of Quantitative Trends: The data summarized in Table 6 provide a general comparison of the performance characteristics of different Ti_3_C_2_T_x_-based coating architectures. Pristine, unshielded Ti_3_C_2_T_x_thin films possess high electrical conductivity, but they are susceptible to rapid degradation in humid environments containing water and oxygen, leading to the formation of insulating TiO_2_ and consequent loss of functional properties [53]. Mechanically, bare films exhibit severe surface pinning (τ_ice_ up to 380 kPa), offering no advantage over standard bare glass-reinforced polymer (GRP) substrates (250–450 kPa). In contrast, embedding the flakes within a cross-linked polymer matrix or shielding them via layered fluorinated topcoats successfully stabilizes the network, yielding greater than 95% conductivity retention under extended humidity, cyclic thermal loads, and intense ultraviolet irradiation. Furthermore, only this stratified layered stack design successfully drops ice adhesion below the critical 15 kPa threshold—directly matching the behavior of passive superhydrophobic reference surfaces—while maintaining stable active electrothermal functionality under convective airflows.

### 8.2. What the Wind Turbine De-Icing Literature Contributes

The wind turbine de-icing literature brings a different but equally important perspective. Studies on electric heating, hybrid coatings, and blade icing make clear that airflow, geometry, and service environment strongly affect de-icing outcome [95]. They also show that energy demand can be lowered through heat-flux management, intermittent activation, and hybrid strategies that combine passive surface design with targeted heating [96,97]. Just as importantly, blade technologies must be assessed at more than one scale, from material coupons to blade sections and ultimately system-level operation [98].

### 8.3. The Translational Gap

MXene anti-icing studies often focus on photothermal or electrothermal response on small samples and may emphasize contact angle, peak temperature, or de-icing videos [99]. Blade-oriented studies, by contrast, are more concerned with aerodynamics, heater control, and energy budgets, but usually rely on conventional conductive materials. Very few reports genuinely integrate MXene materials design with blade-level issues such as leading-edge protection, large-area electrode architecture, lightning coexistence, repair logistics, and maintenance economics.

### 8.4. The Most Important Role of Ti_3_C_2_T_x_ at This Stage

At this stage, MXenes are most credible in the near term, not as stand-alone miracle coatings, but as multifunctional components within hybrid blade-surface systems. Their strongest role is likely to be as conductive and photothermal building blocks embedded in polymer-rich stacks that also provide adhesion, moisture management, oxidation resistance, and erosion tolerance [100,101].

A quantitative comparison of ice-adhesion strength across different coating classes is essential for assessing the practical value of MXene-based anti-icing surfaces. Reported ice-adhesion strength values for MXene-containing coatings typically fall in the range of approximately 20–80 kPa, depending on the matrix polymer, surface chemistry, MXene loading, and measurement protocol [52]. By comparison, bare metallic or composite substrates often exhibit ice-adhesion strengths of several hundred kPa to over 1 MPa, while state-of-the-art superhydrophobic surfaces can reach values below 20 kPa under ideal conditions. However, superhydrophobic performance frequently degrades under repeated icing, condensation, or abrasion [10,12]. Low-ice-adhesion surfaces based on slippery liquid-infused porous surfaces (SLIPS) or low-surface-energy elastomers can also achieve values below 10 kPa, but their durability on eroding blade surfaces remains largely unproven. For blade applications, the more meaningful comparison is not the best-case ice-adhesion value measured on a pristine coupon but the value retained after representative environmental aging, erosion testing, and multiple icing–de-icing cycles. Future studies should therefore benchmark MXene-based coatings against at least three reference conditions: the bare blade substrate material, a superhydrophobic reference coating of comparable thickness, and a commercial or near-commercial low-ice-adhesion coating, all tested under identical ice type, temperature, and measurement conditions.

From a quantitative-stability perspective, the most critical reporting gap in the current MXene anti-icing literature is the lack of systematic conductivity-retention data under combined environmental stressors [69,93]. Key stability metrics that should be reported include: (a) conductivity retention (%) as a function of humidity-exposure time at controlled relative humidity; (b) conductivity retention after accelerated UV irradiation at doses representative of outdoor service; (c) resistance drift after thermal cycling between sub-zero and elevated temperatures for hundreds to thousands of cycles; (d) conductivity retention after freeze–thaw cycling combined with humidity exposure; (e) the effect of encapsulation on all of the above, comparing bare MXene films with encapsulated or buried-MXene architectures; and (f) spatial uniformity of conductivity retention, since localized degradation is often more damaging to heater performance than a uniform small decline in average conductivity. Where the available literature does not report these values, this should be explicitly identified as a data gap rather than implying that stability has already been demonstrated. Such transparency would help the field prioritize the most urgent durability questions and accelerate the development of blade-qualifiable MXene coating systems.

## 9. Key Challenges Before Deployment on Wind Turbine Blades

### 9.1. Oxidation and Storage Stability

**Figure 3:**
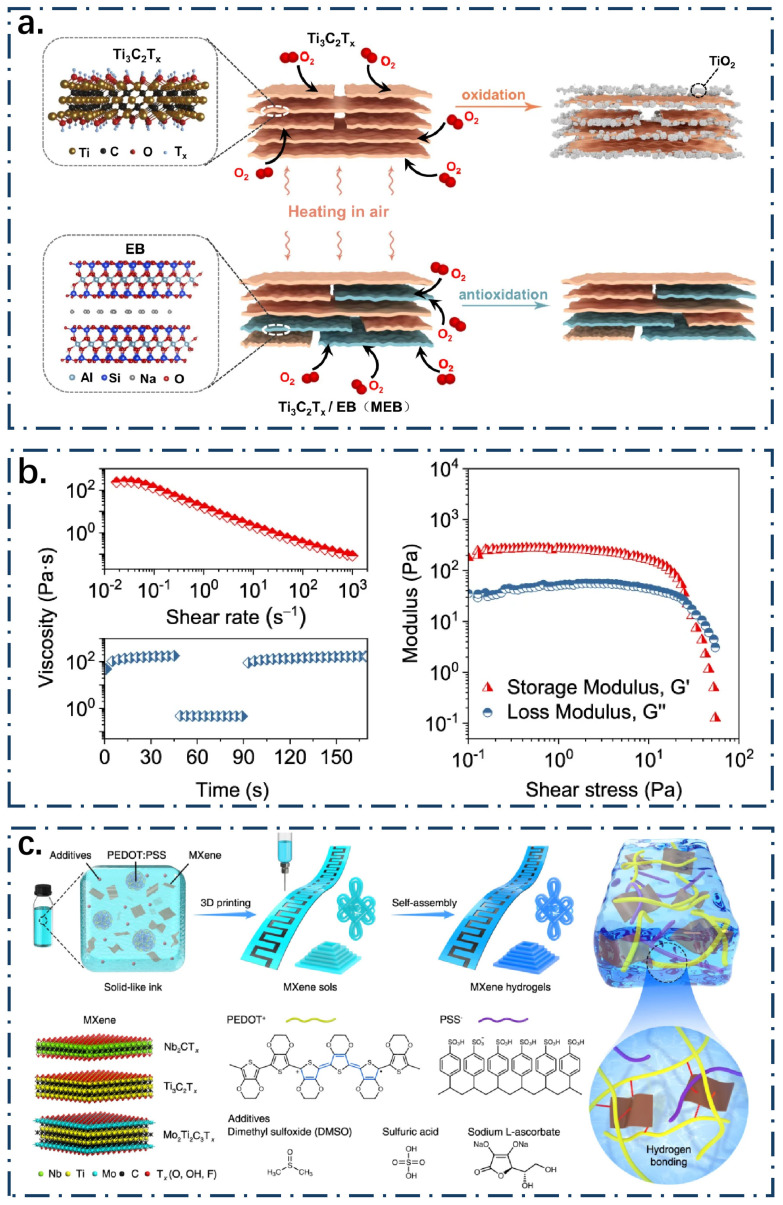
Strategies for oxidation mitigation and scale-up processing of MXene coatings. (**a**) Schematic illustration of structural degradation pathways in air and representative antioxidation or encapsulation approaches [102]. (**b**) Rheological descriptors, including viscosity and viscoelastic response, that govern uniform large-area processing [28]. (**c**) Translation of scalable coating routes into functional large-area Joule-heating elements suitable for structural integration [103].

Oxidation remains the first major obstacle. It affects not only the storage life of MXene dispersions but also the operational lifetime of the final coating [102]. Industrial deployment requires predictable handling, transport, mixing, and application. If conductivity drifts substantially before coating or declines rapidly after installation, the material becomes difficult to justify commercially. More work is therefore needed on stabilized precursor formulations, antioxidant additives, edge passivation, and matrix designs that protect Ti_3_C_2_T_x_ without eliminating the electrical performance required for heating. To better visualize the urgency of these interventions, Figure 3a maps the typical structural degradation pathways of exposed flakes in air, contrasting them with representative antioxidation and encapsulation approaches.

### 9.2. Erosion Resistance Under Real Blade Conditions

Erosion resistance is equally indispensable. Blade surfaces experience repeated high-speed droplet impact that is already challenging for conventional leading-edge protection systems. A MXene-rich layer placed too close to the environment is unlikely to survive for long unless it is carefully shielded. This strongly favors integrated multilayer stacks and careful positioning of the conductive phase. It also suggests that anti-icing specialists and leading-edge-protection researchers need to work much more closely than they usually do today.

### 9.3. Thermal Uniformity and Fault Tolerance at Large Scale

Thermal uniformity at large scale remains another unresolved issue. Meter-scale coatings demand not only homogeneous materials but also robust electrical architecture. Fundamentally, achieving this material homogeneity during large-area processing is governed by the ink’s rheological descriptors, highlighting the critical importance of the viscosity and viscoelastic responses detailed in Figure 3b. Segmented heating zones, fault-tolerant busbar layouts, and dynamic local power control will probably be necessary. Yet, most academic studies are still performed on centimeter-scale samples [88]. Until scale-induced nonuniformity is addressed explicitly, it will remain difficult to judge whether promising laboratory coatings can perform credibly on real blades.

### 9.4. Power Consumption and System-Level Efficiency

Power demand must also be evaluated at the turbine-system level rather than only in terms of areal power density on a coupon. The relevant question is not simply whether a coating can melt ice quickly in the laboratory, but whether the seasonal electrical cost and added system complexity are justified by recovered power generation and reduced downtime. Hybrid strategies that delay icing and heat only critical areas are likely to be more attractive than continuous full-surface heating.

### 9.5. Integration with Existing Blade Systems

Blade surfaces already have to satisfy several functional requirements at once, including erosion protection, lightning management, structural health monitoring, and, in some cases, acoustic or optical sensing. As shown in Figure 3c, MXene materials can be combined with polymers via 4D patterned printing, which facilitates the multifunctional integration and coordinated design of MXene-based systems on wind turbine blades [103]. A MXene-based heater can coexist with these systems. Its conductivity may be beneficial in one context and problematic in another, for example, if it alters lightning-current pathways or interferes with sensors [104]. These questions make multiphysics integration studies essential.

### 9.6. Environmental and Sustainability Considerations

Environmental and sustainability issues should also be addressed more directly. Some MXene synthesis routes still rely on aggressive etching chemistries [105,106,107]. If the target application is wind energy, which is motivated in part by sustainability, then formulation, waste treatment, and end-of-life considerations matter. Similar scrutiny applies to fluorinated low-surface-energy modifiers, which may improve anti-icing performance while creating regulatory or environmental concerns.

### 9.7. Standardization of Tests

Finally, progress is slowed by inconsistent testing protocols. Studies vary widely in substrate type, ice thickness, voltage, humidity, wind speed, and performance criteria, which makes cross-comparison difficult. Blade-oriented benchmarking would benefit greatly from more standardized testing matrices and reporting practices.

## 10. Future Research Directions

### 10.1. Multilayer Stacks Designed from the Outside-In

A productive way to design future blade coatings is to begin with the service environment and work inward, rather than starting from an isolated nanomaterial and adding functions afterward. The outermost layer should first be selected for erosion resistance, ultraviolet stability, water management, and low ice adhesion. Beneath that, barrier and thermal-coupling layers can be introduced, followed by a MXene-rich distributed heater tailored to the available power electronics, and finally a primer optimized for adhesion to the composite substrate. This outside-in logic is more likely to yield deployable systems than simply maximizing conductivity in exposed MXene films.

### 10.2. Smart, Sensor-Coupled and Zonal Anti-Icing Systems

Smart control is another important direction. Because condensate removal, frost suppression, and interfacial de-icing all require less energy than bulk ice melting, early intervention is crucial. MXene-based coatings could be paired with humidity, temperature, impedance, or acoustic sensors, or perhaps exploit changes in their own electrical response as part of a sensing strategy [108]. Zonal activation informed by real-time conditions could significantly improve overall system efficiency.

### 10.3. New MXene Chemistries and Surface Engineering

Although Ti_3_C_2_T_x_ dominates current work, future studies may identify other MXene chemistries with improved oxidation resistance, different optical behavior, or better compatibility with selected matrices. Surface engineering will be particularly important: reactive sites may need to be passivated or modified in ways that preserve conductivity while reducing sensitivity to water and oxygen [109,110,111]. Any such new chemistry, however, should be judged not only by peak laboratory performance but also by reproducibility and manufacturing practicality.

### 10.4. Coupling with Self-Healing and Damage-Tolerant Matrices

Because blade coatings inevitably experience damage, self-healing or damage-tolerant matrices could substantially extend service life. Elastomeric binders, reversible polymer networks, or microcapsule-assisted repair chemistries may help restore barrier properties after minor cracking. Even partial recovery that delays moisture ingress could be highly valuable for MXene-containing layers [112]. When combined with redundant conductive networks, such matrices may enable graceful degradation rather than abrupt heater failure [113].

### 10.5. Integrating Anti-Icing with Other Blade Functions

Multifunctionality is another potential advantage. Ti_3_C_2_T_x_ are already being investigated for thermal management, sensing, electromagnetic shielding, and flexible electronics [114]. On blades, this opens the possibility of coatings that combine de-icing with strain sensing, condition monitoring, or local electromagnetic management. The challenge is to ensure that each additional function strengthens the overall engineering case rather than compromising durability or complexity.

### 10.6. Realistic Pilot Demonstrations

Ultimately, the field will need pilot-scale demonstrations. The next step is not another small coupon that heats efficiently under ideal conditions, but blade coupons and blade sections tested in icing wind tunnels, followed by limited field trials in representative environments. Such studies should report durability, maintenance strategy, failure analysis, and techno-economic considerations alongside thermal performance. Without this level of evidence, MXene-based blade anti-icing will remain scientifically interesting but industrially uncertain.

## 11. Conclusions

Ti_3_C_2_T_x_ represent a promising materials platform for blade de-icing and defogging because they bring together several attributes that are difficult to combine in a single system: high electrical conductivity, strong optical absorption, solution processability, and chemically tunable interfaces [14,15,16,115]. These features are particularly attractive for wind turbine blades, where coatings must remain thin, lightweight, conformal, and functionally integrated with composite structures.

The literature reviewed here indicates that the most plausible route forward is not an exposed MXene film, but a multilayer hybrid architecture in which Ti_3_C_2_T_x_ provide the conductive and photothermal backbone, while polymers, secondary nanocarbons, and protective layers supply adhesion, barrier performance, moisture control, oxidation resistance, and erosion tolerance [116,117]. For blade applications, electrothermal heating should remain the main actuation mode, while photothermal assistance and wettability engineering are better viewed as ways to lower the energy burden rather than replace active heating. Defogging also deserves more attention because early control of condensate can prevent progression toward frost and ice.

MXenes are therefore not yet a turnkey solution for wind turbine blades, but they are among the most promising building blocks for lightweight, low-profile, and multifunctional anti-icing systems. The field now needs to move beyond proof-of-concept heating demonstrations toward stability engineering, erosion-resistant stack design, large-area current management, smart control, and pilot-scale validation. If those challenges are addressed, MXene-based coatings could become a useful enabling technology for reliable all-weather wind-energy operation.

## Figures and Tables

**Table 1:** Critical design dimensions of wind turbine blades.

Dimension	Key Message	Role
Materials	Ti_3_C_2_T_x_ provide high conductivity, broadband light absorption, interfacial tunability, and compatibility with polymer matrices.	Enables heater layers and multifunctional coatings that remain thin, lightweight, and conformable.
Functional route	Electrothermal heating is the most blade-relevant route, while photothermal and wettability engineering can lower energy demand.	Hybrid active–passive strategies are more realistic than purely passive or purely photothermal designs.
Engineering bottlenecks	Oxidation, erosion, thermal non-uniformity, adhesion loss, and large-area manufacturing remain unresolved.	Blade deployment requires coating systems that survive rain, sand, salt, UV, freeze–thaw cycling, and mechanical loading.

**Table 2:** Benchmarking matrix for emerging nanomaterial coating systems.

Evaluation Metric	Ti_3_C_2_Tx MXene	Graphene/CNTs	Silver Nanowires
Conductivity (S·cm^−1^)	10^3^ to 2 × 10^4^	10^2^ to 10^4^	10^4^ to 10^5^
Thickness Penalty	Ultrathin (<10 μm)	Thin (<50 μm)	Ultrathin (<5 μm)
Processability	High (Water ink)	Moderate (Surfactant)	High (Solution dispersion)
Oxidation Stability	Poor (TiO_2_ conversion)	Excellent (Inert plane)	Moderate (Sulfidation)
Conformability	High	High	High (Sliding junctions)
Primary Bottleneck	Moisture oxidation	High percolation	Contact migration
Target Blade Role	Electro-photothermal zone	Anti-corrosion barrier	Localized sensing

**Table 3:** Benchmarking matrix for conventional and polymer-based heaters.

Evaluation Metric	Carbon Fiber (CF)	Metallic Mesh/Foil	Conductive Polymer
Conductivity (S·cm^−1^)	10^2^ to 10^3^	10^5^ to 10^6^	10^−1^ to 10^2^
Thickness Penalty	Thick (>1mm)	Thick (>0.5mm)	Thin (<20 μm)
Processability	Low (Prepreg molding)	Very Low (Fixed profile)	High (Solution process)
Oxidation Stability	Excellent (Inert carbon)	Moderate (Corrosion)	Poor (UV aging/de-doping)
Conformability	Low (Rigid composite)	Very Low (Fatigue prone)	Very High
Primary Bottleneck	High thermal mass	Discrete hotspots	Dynamic doping loss
Target Blade Role	Primary spar heater	Non-aerodynamic areas	Antistatic shield

**Table 4:** Representative design considerations for MXene-enabled blade systems.

Design Variable	Typical Options	Benefit
Conductive phase	Pure MXene; MXene/CNT; MXene/graphene; MXene/conducting polymer	Tailors resistance, flexibility, and redundancy
Matrix/binder	PU, epoxy, silicone, fluorinated polymer, elastomer	Adhesion, barrier protection, toughness, weatherability
Architecture	Single layer, bilayer, multilayer, gradient stack	Separates heater, barrier, and low-ice-adhesion functions
Placement	Near-surface heater or buried heater below topcoat	Balances rapid response and environmental protection
Activation mode	Electrothermal; electrothermal + photothermal; zonal/intermittent control	Reduces energy consumption and improves adaptability

**Table 5:** Qualitative benchmarking of blade-heating technologies for wind turbine anti-icing.

Technology	Weight Penalty	Integration Complexity	Power Demand	Repairability	Key Deployment Barriers
MXene-based thin coatings	Low (<200 g/m^2^)	Moderate (new material, electrodes needed)	Low–moderate (zonal, interfacial)	Potentially good (local recoating feasible)	Oxidation stability, erosion resistance, scale-up, lightning compatibility, lack of long-term field data
Metallic resistance heaters (wires, foils, meshes)	Moderate–high	Moderate (mature, but integration into composite required)	Moderate–high (bulk heating)	Difficult (embedded, requires section replacement)	Weight, fatigue mismatch, corrosion, thermal expansion mismatch
Carbon-fiber or carbon-based heaters	Low–moderate	Moderate (can be integrated into composite layup)	Low–moderate	Difficult (embedded)	Anisotropic conductivity, oxidation of carbon above ~400 °C, contact resistance, cost of high-quality carbon fabrics
Conductive paints/coatings (non-MXene)	Low–moderate	Low (sprayable, familiar application)	Moderate (higher resistance, less uniform)	Good (resprayable)	Lower conductivity and heating efficiency, durability under erosion, filler settling, resistance drift
Hot-air systems	Low (no coating on blade)	High (ducting, blowers, heaters in nacelle/hub)	High (heating entire blade cavity)	System-level maintenance	Poor energy efficiency, slow response, thermal gradients, noise, limited to internal heating

**Table 6:** Quantitative benchmarking and stability profiles of reported Ti_3_C_2_T_x_ anti-icing systems.

Evaluation Metric & Physical Baselines	Pure/Bare Ti_3_C_2_T_x_ Thin Films	Carbon-Hybrid Networks (Ti_3_C_2_T_x_/CNT or rGO)	Layered Stacks/Polymer Encapsulated
Sheet Resistance (Rs)	0.5 to 25 Ω/sq	8.0 to 85 Ω/sq	15 to 350 Ω/sq
Applied Voltage Range	1 to 12 V	5 to 24 V	12 to 48 V
Areal Power Density (Pareal)	0.2 to 3.5 W·cm^−2^	0.15 to 2.2 W·cm^−2^	0.1 to 1.8 W·cm^−2^
Heating Rate (Still Air)	15 to 45 °C/s	5 to 18 °C/s	1 to 8 °C/s
Steady-State Temp. under Airflow	35 °C to 65 °C (at 10–15 m/s) (Still air ref: 100–180 °C)	30 °C to 55 °C (at 10 m/s) (Still air ref: 70–120 °C)	25 °C to 50 °C (Under convective airflow) (Still air ref: 50–95 °C)
Ice Adhesion (τ_ice_) vs. Reference Surfaces	τ_ice_ = 220 to 380 kPa·Bare GRP substrate ref: 250–450 kPa·Superhydrophobic ref: <20 kPa	τ_ice_ = 110 to 190 kPa·Bare GRP substrate ref: 250–450 kPa·Superhydrophobic ref: <20 kPa	τ_ice_ < 15 kPa·Bare GRP substrate ref: 250–450 kPa·Superhydrophobic ref: <20 kPa
Quantitative Stability:Conductivity Retention (%)	Humidity: <20% (14 days at >85% RH) UV Dose: <40% (at 100 kJ·cm^−2^)·Thermal Cycles: <50% (after 50 cycles)	Humidity: 75% to 85% (30 days at 90% RH) UV Dose: ~80% (at 200 kJ·cm^−2^) Thermal Cycles: >88% (after 200 cycles)	Humidity: >95% (90 days at 95% RH) UV Dose: >90% (at >500 kJ·cm^−2^) Thermal Cycles: >96% (after 500 cycles)
Primary Engineering Reporting Gaps	Omits convective heat loss coefficient (h_c_)	Unspecified dynamic structural fatigue limits	Lacks absolute rain erosion mass loss rate

## Data Availability

No new data were generated or analyzed in this study. Data sharing is not applicable to this article.
